# Paracetamol as a toxic substance for children: aspects of legislation in selected countries

**DOI:** 10.1186/s12995-015-0084-3

**Published:** 2015-12-10

**Authors:** Menen E. Mund, David Quarcoo, Christoph Gyo, Dörthe Brüggmann, David A. Groneberg

**Affiliations:** Departments of Female Health and Preventive Medicine, Institute of Occupational Medicine, Social Medicine and Environmental Medicine, Goethe University, Frankfurt am Main, Theodor-Stern-Kai 7, Frankfurt, 60590 Germany; Department of Obstetrics and Gynecology, Keck School of Medicine of USC, Los Angeles, California USA

**Keywords:** Paracetamol, Acetaminophen, Analgesics, Poisoning, Intoxication, Child, Pediatrics, Legislation, European Union, Non-pharmacy

## Abstract

Paracetamol is used widely in pediatrics because it has a high drug safety when used in therapeutic dosages. In case of overdose the majority of paracetamol is metabolized to N-acetyl-p-benzoquinone imine (NAPQI), which is responsible for the severe toxic effects. The covalent connection between NAPQI and hepatic proteins leads to hepatocellular damage and possibly to severe liver failure. The antidote for paracetamol is N-acetylcysteine (NAC). It is a precursor of glutathione and aids to fill glutathione stores. The Rumack-Matthew nomogram should be used to decide on antidote treatment. Pediatric drug metabolism differs from adult metabolism. Children have a larger liver size compared to their body weight than adults, resulting in a higher metabolism rate. Young children seem to be less sensitive to acute intoxication than adults. One hypothesis to explain the lower rate refers to the larger liver size. The acute toxic dosage for children is more than 200 mg/kg body weight. There seems to be a global increase in accidental pediatric paracetamol overdose. Governmental websites of various European Union (EU) countries were searched for legal information on paracetamol availability in pharmacies and non-pharmacy stores. Various EU countries permit prescription-free sales of paracetamol in pharmacies and non-pharmacy stores. In Sweden paracetamol 500 mg may be sold in both pharmacies and non-pharmacies in a maximum pack size of 20 units. In the United Kingdom (UK) paracetamol 500 mg is listed in the general sales list with a maximum pack size of 30 effervescent tablets or 16 tablets. In Ireland paracetamol 500 mg may be sold in a maximum pack size of 12 units in a non-pharmacy. In the Netherlands paracetamol 500 mg is legal to be sold in a maximum pack size of 50 units in a drug store and with a maximum of 20 units in any other non-pharmacy. Several countries in the European Union are permitted to offer paracetamol prescription-free in pharmacies and non-pharmacy stores without legal guidance on the storage position within the store. Further research is needed to investigate whether paracetamol is located directly accessible to young children within the stores in EU countries which permit prescription-free sales of paracetamol.

## Background

**Fig. 1:**
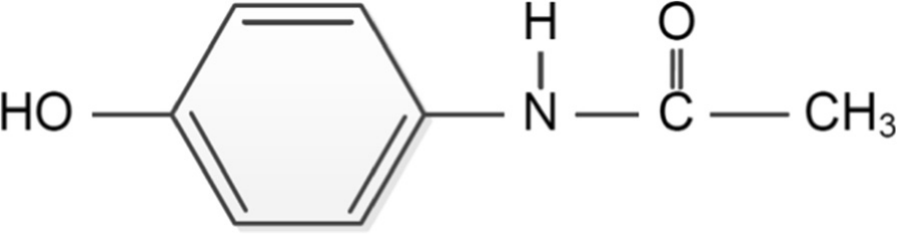
Chemical structure of paracetamol, also known as N-acetyl-p-aminophenol, modified after [39]

Paracetamol is an important drug against pain and fever. It is highly popular and used worldwide. Paracetamol has a high drug safety when used in recommended dosages. In the United States of America (US) approximately 50 million adults consume paracetamol containing products every week. Nevertheless, an overdose can lead to severe liver failure [1]. The substance is named differently throughout the world; in the US and Canada the name *acetaminophen* is mostly used. In other countries like Germany, the UK or Australia it is called *paracetamol*. Both names have their origin from the labeling of the chemical structure [2] (Fig. 1). This narrative review summarizes the effects of paracetamol intoxication, especially in children. Additionally, it outlines the current legal requirements on paracetamol sales in various countries of the European Union in order to establish whether the pharmaceutical might be accessible to young children within pharmacies and non-pharmacy stores.

## Pharmacological differences between children and adults

Some fundamental pharmacological effects differ between children and adults. Additionally, differences occur between the diverse pediatric age groups. Table 1 provides an overview of age subdivision in pediatric population. The largest variations exist between neonates and adults. Drug absorption depends on assimilation route and biochemical drug characteristics. Most pharmaceuticals in pediatrics are administered orally and therefore gastric pH and gastrointestinal motility are important. Drug absorption does not seem to differ significantly between infants, older children or adults [3, 4]. Metabolism of a drug is often dependent on the liver. Metabolism rate depends on two factors: the size of the liver and the effectiveness of enzymes. Children have a much larger liver size compared to their body weight than adults; liver volume relative to body weight is twice as high in a child aged 1 year than in a child aged 14 years. Subsequently, children have a higher metabolism rate than adolescents and adults. One exception is neonates as their enzyme production capacity is still immature and cannot function as effectively yet. Renal elimination is an important way of drug elimination. Tubular secretion and reabsorption are immature at birth, but they reach maturity during the first year of life and achieve adult capacities [3, 4].

**Table 1:** Subdivision of pediatric population in age groups, modified after [40]

Pediatric group	Age
Neonates	Birth to 1 month
Infants	1 month – 2 years
Young Children - Preschool	2 years – 6 years
Older Children - School	6 years – 12 years
Adolescents	12 years – 18 years

## Paracetamol toxicity

A US study from 2011 showed that both intentional and accidental paracetamol poisoning continues to be a major public health problem [5]. Paracetamol-induced hepatotoxicity is the most common cause of liver failure in the US [6]. In adults it was demonstrated that self-treatment with paracetamol containing pharmaceuticals can lead to unintentional poisoning [7]. According to the pharmacologist *K. Brune* paracetamol belongs to the most hazardous substances throughout the whole world [8]. The hepatotoxic effect of paracetamol was first described in 1966 by *D.G. Davidson* and *W.N. Eastham*. They reported on two patients who had died of fulminate liver necrosis in the centrilobular areas after paracetamol overdose. Since then numerous studies have emphasized the hepatic toxicity of this substance [1]. Paracetamol is metabolized via three different pathways: conjugation with sulfate, conjugation with glucuronide and transformation with the cytochrome P450 enzyme system to the highly active metabolite N-acetyl-p-benzoquinone imine (NAPQI) [9]. The metabolite NAPQI is responsible for the severe toxic effects. When used in therapeutic dosage paracetamol is mainly metabolized via the first two pathways; only 5 % of the substance is transformed to NAPQI [9]. NAPQI is immediately conjugated with reduced glutathione to form a hazard-free metabolite [10]. However, in case of overdose the first two pathways become saturated and the majority of paracetamol is metabolized to NAPQI. The conjugation between NAPQI and glutathione leads to a decrease of glutathione stores. Subsequently, reduced glutathione cannot be reestablished as efficiently as it is needed to transform NAPQI into a harmless metabolite. High amounts of NAPQI remain unbound which results in covalent binding between free NAPQI and hepatic proteins. This covalent connection leads to hepatocellular damage [11].

Four stages of paracetamol intoxication are described (Table 2) [11, 12]. At stage one, symptoms occur shortly after poisoning and are unspecific. They often include nausea, vomiting and malaise. After one to two days the initial clinical findings diminish, but abdominal pain may occur. In this stage abnormal laboratory values are often observed, including elevated liver enzymes, increased bilirubin and prolonged prothrombin time. Stage three consists of the reappearance of initial unspecific symptoms. In addition, liver function parameters reach high abnormalities. Stage four occurs within the following four to fourteen days; during this time the outcome of the intoxication will appear. The patient will either recover or develop complete liver failure [11]. Even though it is uncommon, cases of acute renal failure without the development of liver failure have been reported [13, 14].

**Table 2:** Stages of paracetamol intoxication, modified after [11, 12]

Stage	Time frame	Symptoms
1	0.5 – 24 h	Unspecific symptoms like diarrhea, nausea, malaise
2	24 – 48 h	Abdominal pain, abnormal laboratory values
3	3 – 4 days	Peak of hepatic dysfunction, stage 1 symptoms may reappear
4	4 – 14 days	Recovery or complete liver failure

## Paracetamol intoxication in children

Paracetamol is the most widely used medication in pediatrics against pain or fever [11]. It is administered in all pediatric age groups from premature neonatal patients to adolescents. For adult usage it is often produced in blister package containing 500 mg tablets. For children various application methods and strengths exist [15, 16]. Three main ways of ingestion lead to paracetamol intoxication in children; deliberate overdose, unintentional exposure or administration error [17]. Because of its popularity in pediatrics, children are frequently used to taking the drug. Adult and children paracetamol medication is often stored at home and might be easily available for children, risking the possibility for unintentional poisoning in children by ingesting the pharmaceutical [11].

The acute toxic dosage for children appears to be more than 200 mg/kg body weight. In case of repeated dosage, intoxication seems to occur after application of more than 75 mg/kg body weight a day in children younger than 6 years of age. Acute toxic dose in adolescents is considered to be more than 7.5 g in a single dose [11, 18], even though in different ethical groups like Japanese lower doses might already lead to intoxication [19]. Nevertheless, children seem to be less sensitive to acute intoxication than adults. One hypothesis to explain the lower rate of severe intoxication in children in comparison to adults refers to their larger liver size in relation to body weight. Subsequently, children are probably able to metabolize paracetamol more effectively than adults because of larger glutathione stores [20].

The real incidence of liver failure in pediatric patients due to paracetamol intoxication remains uncertain. A Spanish study researched the incidence of oral antipyretic poisoning in children up to 14 years. Out of all cases, 11 % occurred due to paracetamol poisoning of which 9.4 % were severe [21]. The US and the UK report paracetamol intoxication to be the main cause of pediatric liver failure, accounting for 14 % of all cases. Most of these poisonings are due to intentional overdose in adolescents, but there seems to be a global increase in accidental overdose in children [17].

## Treatment of acute paracetamol intoxication

**Fig. 2:**
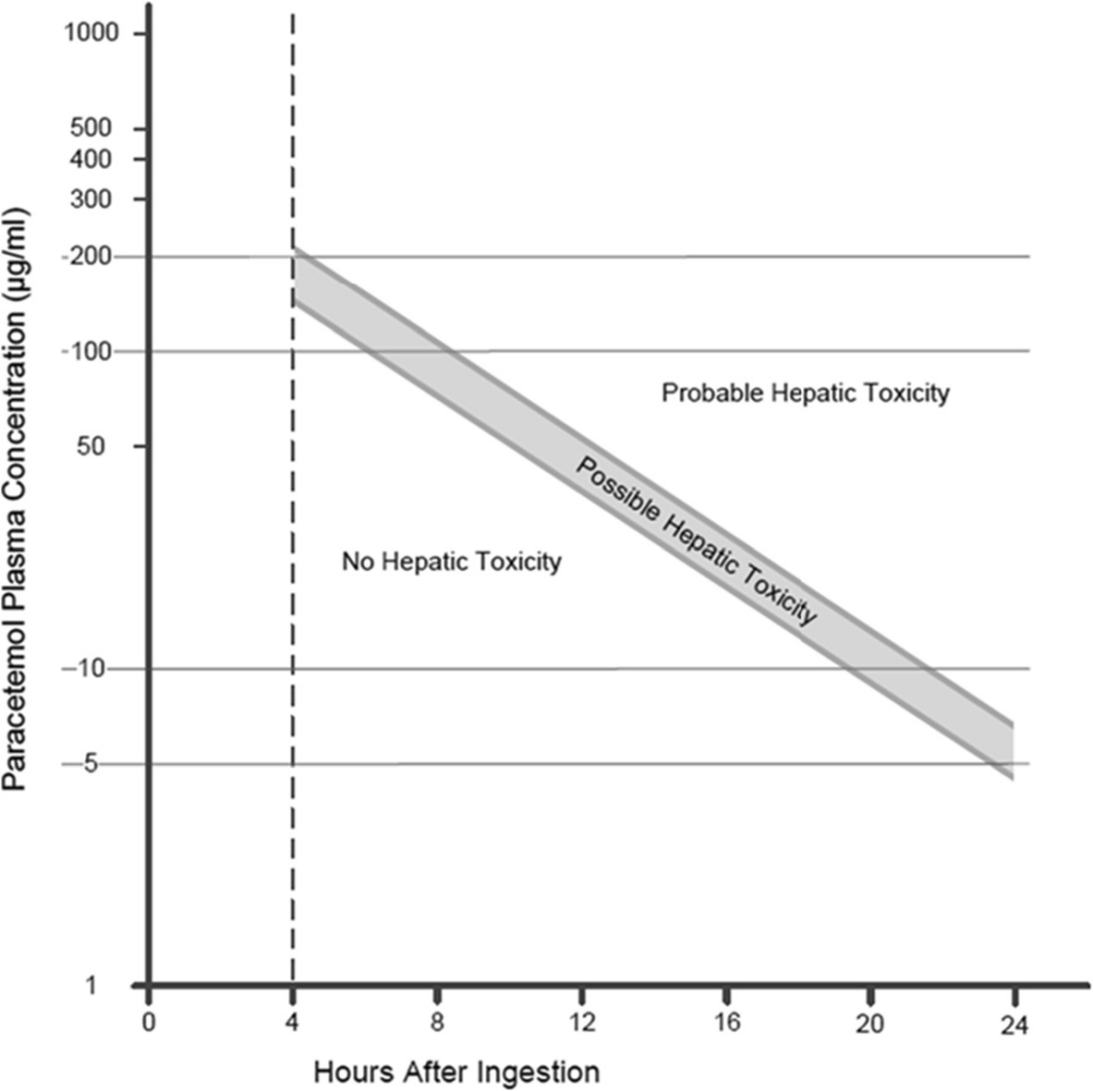
Rumack-Matthew nomogram, modified after [11]

Immediate identification of patients with acute paracetamol intoxication is essential to reduce morbidity and mortality [12]. Activated charcoal should be administered instantly after acute ingestion [22]. The antidote for paracetamol is N-acetylcysteine (NAC). It is a precursor of glutathione and aids to fill glutathione stores; full glutathione stores prevent the reaction of unbound NAPQI with hepatic cells. It does not affect the paracetamol concentration or its elimination. It is important for the treatment to start as soon as possible after poisoning [11]. The Rumack-Matthew nomogram should be used to decide on antidote treatment (Fig. 2). The nomogram was first established by *B.H. Rumack* and *H. Matthew* on basis of a retrospective study of 64 cases of acute paracetamol ingestion. It exhibits the correlation between paracetamol blood concentration and hepatocellular injury. Hepatic toxicity is predicted in patients with paracetamol blood concentration at or above a threshold line between 200 and 6.25 μg/ml 24 h post ingestion. After the nomogram was introduced in the US, the US Food and Drug Administration (FDA) insisted on lowering the treatment threshold by 25 % for safety reasons. Consequently, a line between 150 and 4.7 μg/ml 24 h post ingestion is considered as the treatment threshold. NAC therapy should be initiated in patients with paracetamol levels at or above this threshold [12]. Paracetamol concentration should be determined from 4 h on after ingestion. Measurements prior to this time frame may not reveal peak concentration and therefore might be incorrect [11].

NAC has a high benefit. The antidote treatment is considered efficient as it reduces the mortality to <0.5 % even after serious hepatotoxicity. The effectiveness of NAC therapy is not reduced by the hours passed after poisoning or the heaviness of symptoms. Different concepts exist on NAC therapy; two main protocols recommend a 20-h intravenous treatment or a 72-h oral treatment. Both approaches are well studied and considered effective and safe. Oral administration may irritate the gastrointestinal tract and can lead to vomiting; it should be a patient specific choice which method of application to use. Liver transplantation is the only option left if NAC treatment failed in patients with severe hepatic injuries after paracetamol intoxication [11, 12, 23].

## Pharmaceutical legislation in European Union countries

European Union countries receive legislation from two different legislative authorities. On the one hand, national governments enact laws which apply to every distinct country individually. On the other hand, laws in EU countries can be enacted by the EU. The European Commission (EC), the European Parliament (EP) and the European Council form the EU legislative body. The EC proposes legislation which is discussed and eventually enacted by the EP and the European Council. Two different types of legislation are performed by the EU: regulations and directives. An EU regulation is instantly enforced as law in all EU member country at the same time; it excels national law. EU directives are guidelines with a defined goal and time period in which it has to be incorporated into national law. Legislation processes on EU level are complicated; occasionally it takes long time until a regulation or directive becomes enforced [24–26]. The EU pharmaceutical law consists of diverse regulations and directives concerning numerous pharmaceutical subjects like pharmacovigilance, falsified medical products, drug marketing and regulatory processes. Accessibility of the pharmaceuticals paracetamol however is not regulated by European law but by national law [27]. For this narrative review governmental websites of various EU countries were searched in order to analyze legal information on paracetamol availability in pharmacies and non-pharmacy stores. Four countries which have been part of the EU before 1995 were selected. Sweden was chosen as a Scandinavian country. Both Ireland and the UK were chosen due to their proximity on the British Isles. The Netherlands represent a Benelux country. The aim was to represent a cultural variety within Western European countries.

## Legislation in Sweden

The *Swedish Medicines Act* enables the *Medical Product Agency* to control and monitor medical items in Sweden. The agency is furthermore entitled to classify drugs into prescription-free and prescription-only medication [28, 29]. Purchase without prescription is allowed for paracetamol under certain circumstances (Table 3). Paracetamol is allowed to be sold prescription-free in strength of 20 units of 500 mg in both pharmacies and non-pharmacies [30].

**Table 3:** Paracetamol availability in Sweden, modified after [30, 41–43]

Application form	Dosage per unit	Pack size non-pharmacy	Pack size pharmacy
Tablet	250 mg	20 units	20 units
Tablet	500 mg	20 units	20 units
Oral solution	24 mg/ml	100 ml	100 ml
Suppository	500 mg	10 units	10 units

## Legislation in the United Kingdom

The *Medicines and Healthcare Products Regulatory Agency* is an administrative body of the British *Department of Health*; this agency is responsible for admission, monitoring and safety of pharmaceuticals in the United Kingdom. The *Medicines Act* from 1968 constitutes the legislation of pharmaceuticals. Medicines are divided into three different categories:

- Prescription-only medication
- Pharmacy sales medication
- General sales list medication (GSL)

Certain requirements in package size and strength apply for paracetamol in order to be listed as GSL (Table 4). Maximally 30 effervescent tablets (TEF) or 16 tablets (tab) or capsules of paracetamol 500 mg may be offered in non-pharmacy stores [31, 32].

**Table 4:** Requirements for paracetamol to be listed GSL in the UK, modified after [32]

Application form	Dosage per unit	Pack size
Tablet or capsules	500 mg (adults) 120 mg (children)	16 tablets or capsules
		30 effervescent tablets
Powders or granules	1000 mg (adults) 240 mg (children)	10 units
Liquids	5 %	160 ml

## Legislation in Ireland

The *Irish Medicines Board* forms the official agency of the *Department of Health and Children.* This agency regulates pharmaceutical matters like drug safety, drug risks and drug monitoring. The *Medical Product Regulations Law* regulates prescription and supply of pharmaceuticals. Three categories for medication exist in Ireland. Pharmaceuticals are either classified as:

- General sales medication
- Prescription-controlled medication from *schedule 1* (S1)
- Exemption from S1 medication.

These exemptions are either pharmaceuticals listed in *schedule 2* (S2) or S1 pharmaceuticals which are labeled with maximum dose, maximum daily dose or maximum treatment period. Paracetamol availability is regulated with particular restrictions in Ireland; caution declarations must appear on the package to give warning of overdose. It is only allowed to purchase one single package at a time. In certain maximum strengths and package sizes paracetamol is available in non-pharmacy stores [33–35] (Table 5).

**Table 5:** Paracetamol availability in Ireland, modified after [35]

Application form	Dosage per unit	Pack size non-pharmacy	Pack size pharmacy
Tablet/capsule/sachet	500 mg	12 units	24 units
Tablet/capsule/sachet	600 mg	10 units	20 units
Tablet/capsule/sachet	1000 mg	6 units	12 units
Liquid	50 mg/ml	60 ml	240 ml

## Legislation in the Netherlands

The Dutch medicine law came into effect in 2007 and was enforced by the *Ministry of National Health, Wellbeing and Sports*. This ministry instructs the administrative body *Medicines Evaluation Board* to control pharmaceutical safety and quality. Medications are divided into four different categories in the Netherlands [36]:

- Prescription-only medication (UR)
- Pharmacy-only medication (UA)
- Pharmacy or drug store-only (UAD)
- Open sales medication (AV)

Paracetamol differs in classification according to strength and application form (Table 6). Paracetamol has AV status until a package size of 20 tablets of 500 mg and subsequently can be sold in any shop. Package sizes of 20 – 50 tablets of 500 mg have UAD status and may only be sold in drug stores or pharmacies [37, 38].

**Table 6:** Classification of paracetamol in the Netherlands, modified after [38]

Application form	Dosage per unit	Pack size	Status
Tablet	500 mg	20 tab	Open sale medication
Tablet	500 mg	20 – 50 tab	Pharmacy or drug store only
Suppository	1000 mg	10 units	Open sale medication

## Conclusions

Paracetamol is a drug which is widely used in pediatrics. Severe intoxication in children seems to be less frequent compared with adults. Nevertheless, paracetamol poisoning can lead to liver failure with possible fatal development in children. Several countries in the European Union are permitted to offer paracetamol prescription-free in pharmacies and non-pharmacy stores without legal guidance on the storage position within the store. If the pharmaceutical is placed openly and within the direct reach of a child within the store, the infant or young child might easily grab the package. This could lead to accidental poisoning if the child swallows the content. Additionally, storing the pharmaceuticals in an unsafe location in a public store might downplay the danger of accidental poisoning and may mislead parents or caregivers to also store the drugs in an unsafe location at home. Accordingly, further investigation is needed to analyze whether the pharmaceutical is placed in direct accessibility to infants and young children within these stores.

## Abbreviations

AV: Open sale medication *(algehele verkoop geneesmiddel)*
EC: European Commission
EP: European Parliament
EU: European Union
FDA: US Food and Drug Administration
GSL: General sales list
NAC: N-acetylcysteine
NAPQI: N-acetyl-p-benzoquinone imine
S1: Schedule 1
S2: Schedule 2
Tab: tablets
TEF: Effervescent tablets
UA: Pharmacy only medication *(uitsluitend apotheek geneesmiddel)*
UAD: Pharmacy or drug store only *(uitsluitend apotheek of drogis geneesmiddel)*
UK: United Kingdom
UR: Prescription only medication (*uitsluitend recept geneesmiddel)*
US: United States of America
